# Iatrogenic Transient Complete Heart Block in a Preexisting LBBB

**DOI:** 10.1155/2016/9531210

**Published:** 2016-07-12

**Authors:** Adil S. Wani, Adebayo Fasanya, Prachi Kalamkar, Christopher A. Bonnet, Omer A. Bajwa

**Affiliations:** ^1^Department of Medicine, Allegheny General Hospital, Pittsburgh, PA 15212, USA; ^2^Department of Pulmonary-Critical Care, Allegheny General Hospital, Pittsburgh, PA 15212, USA; ^3^Department of Medicine, Conemaugh Memorial Medical Center, Johnstown, PA 15905, USA; ^4^Department of Clinical Cardiac Electrophysiology, Allegheny General Hospital, Pittsburgh, PA 15212, USA

## Abstract

Catheter induced cardiac arrhythmia is a well-known complication encountered during pulmonary artery or cardiac catheterization. Injury to the cardiac conducting system often involves the right bundle branch which in a patient with preexisting left bundle branch block can lead to fatal arrhythmia including asystole. Such a complication during central venous cannulation is rare as it usually does not enter the heart. The guide wire or the cannula itself can cause such an injury during central venous cannulation. The length of the guide wire, its rigidity, and lack of set guidelines for its insertion make it theoretically more prone to cause such an injury. We report a case of LBBB that went into transient complete heart block following guide wire insertion during a central venous cannulation procedure.

## 1. Introduction

Central venous cannulation is indispensable in the management of severely ill patients. A number of complications are known to arise from injury to the vein and the neighboring structures. A right bundle branch injury is one of the complications that can occur during the guide wire insertion. This type of injury can go unrecognized but in the presence of a preexisting left bundle branch block (LBBB) it can be life-threatening. We present a case of transient complete heart block that was caused during a guide wire insertion in a patient with a preexisting LBBB and discuss the incidence of such a complication and possible ways to prevent it.

## 2. Case Description

**Figure 1:**
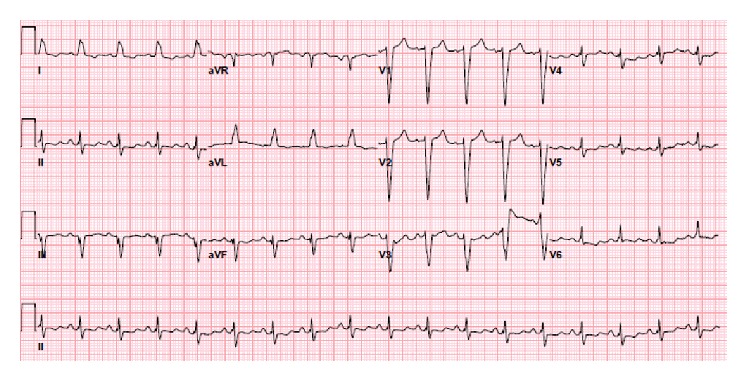
EKG at admission showing the preexisting left bundle branch block.

**Figure 2:**
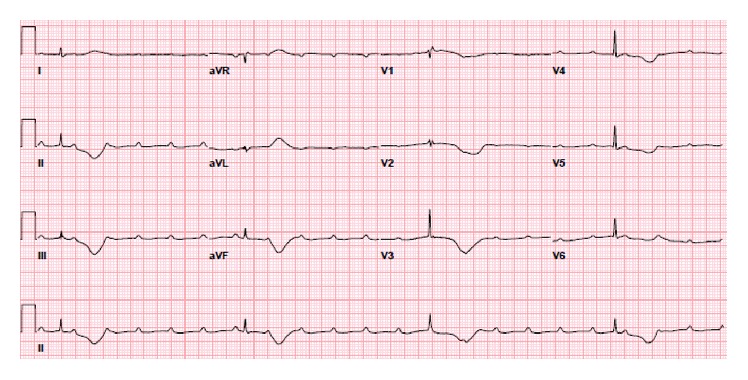
EKG following the procedure showing a complete heart block with an escape rhythm.

A 77-year-old woman was transferred to the intensive care unit (ICU) of our hospital with altered mental status that was thought to be secondary to carbon dioxide retention caused by aspiration pneumonia. In the ICU, she progressed into hemodynamic shock. A central venous cannulation was attempted via the right internal jugular vein. Before performing the central venous cannulation, her heart rate was averaging 70–80 beats per minute. Following the cannulation, her heart rate suddenly dropped to 20 beats per minute and the cardiac tracing on the monitor showed a complete heart block (CHB). A stat electrocardiogram (EKG) was obtained which confirmed complete heart block with an escape rhythm (Figure 2). Atropine was administered which failed to correct the heart rate. A chest radiograph showed the tip of the catheter in the superior vena cava. A transcutaneous pacemaker was immediately placed followed by a temporary transvenous pacemaker placement. Her previous EKG revealed a preexisting left bundle branch block (LBBB) (Figure 1). 24 hours following this event, the CHB resolved and she was back in sinus rhythm with a LBBB. The transvenous pacemaker was removed. An echocardiogram showed normal left ventricular function with an ejection fraction of 55%. Since the CHB was transient and was thought to be due to mechanical inhibition of the right conduction system caused by guide wire insertion, further electrophysiologic studies were not felt warranted. Following this, she had a complicated medical course but she eventually recovered and was discharged in a stable condition without a pacemaker.

## 3. Discussion

A right bundle branch block (RBBB) occurring during the passage of guide wire or a catheter into the heart has an incidence of 3–12% [1–4]. In patients without a preexisting left bundle branch block (LBBB), this complication may go unnoticed. But even a transient RBBB in a patient with a preexisting LBBB can cause hemodynamic compromise due to a complete heart block (CHB) with unstable escape rhythm or even asystole [1]. Studies have shown an increased risk of CHB during a pulmonary artery catheterization in patients with preexisting LBBB. Some authors even suggest a prophylactic temporary pacemaker in patients with preexisting LBBB undergoing a right heart catheterization taking into account the life-threatening consequence of CHB [1, 5, 6] while others argue against it as the small chance of this complication does not warrant a delay in the procedure [2, 3].

The superficial position of the right bundle branch in the right ventricle just below the tricuspid valve makes it more prone to injury from a foreign object [2, 4, 7]. A new anterior or posterior fascicular block along with a RBBB has also been reported, thought to be due to the longitudinal dissociation of fibers in the bundle of His, but the exact mechanism remains unclear [1, 4]. The left bundle is less susceptible due to its early branching in the septum. RBBB along with an anterior fascicular block has also been reported with a left heart catheterization [8].

When compared to pulmonary artery catheterization, a conduction block during central venous cannulation is rare; however unlike the pulmonary artery catheter insertion, a central venous cannulation is not always performed under cardiac monitoring. Moreover the guide wires used during central venous cannulation are more rigid making them theoretically more arrhythmogenic [1]. They are much longer than the actual cannula making them more prone to enter the right ventricle when inserted inadvertently. The mean distance from the insertion site to the junction of superior vena cava with the right atrium is 18 cm with right internal jugular being the shortest (16 cm) and the left subclavian being the longest (21.2 cm) [9]. A prospective study showed the incidence of advanced ventricular arrhythmia to be 20% with an inadvertent insertion of the guide wire to the right ventricle. The incidence of ventricular arrhythmia during a guidewire insertion almost approaches the incidence during a right heart catheterization [10]. Careful insertion of guide wire to less than 22 cm decreased the incidence of complications by 70% [1]. The usual upper limit for safe guide wire insertion in an adult is considered to be 18 cm [9]. This goal can be easily missed especially when the dilator is threaded over the guide wire, and hence withdrawal of the guide wire to control the distal tip is recommended [10].

This case emphasizes the risk of a life-threatening complication that can occur during a guide wire insertion in a patient with preexisting LBBB. While the insertion of a pulmonary artery catheter is usually done in a monitored setting, central venous cannulations are mostly performed in a nonmonitored environment. Ideally a guide wire should have external markings indicating the length in centimeters for better guidance. Many institutions have replaced their central venous insertion kit with a guide wire marked at every 10 cm, but it is yet to be universally accepted. We suggest EKG monitoring for all patients prior to central venous cannulation. Additionally in patients with a preexisting LBBB, one should be very cautious during guide wire insertion, and if time permits, having a noninvasive transcutaneous pacemaker at bedside might be beneficial.
